# Variation in seed properties and germination capabilities among populations of the invasive weed *Parthenium hysterophorus* L. (Asteraceae)

**DOI:** 10.3389/fpls.2023.1222366

**Published:** 2023-07-27

**Authors:** Sahar Malka, Hanan Eizenberg, Maor Matzrafi

**Affiliations:** ^1^ The Robert H. Smith Faculty of Agriculture, Food and Environment, The Hebrew University of Jerusalem, Rehovot, Israel; ^2^ Department of Plant Pathology and Weed Research, Agricultural Research Organization – Volcani Institute, Newe-Ya’ar Research Center, Ramat Yishai, Israel

**Keywords:** cardinal temperature model, germination, maternal effects, transgenerational effects, water potential

## Abstract

**Introduction:**

*Parthenium hysterophorus* (Asteraceae) is an invasive weed species that has invaded over 50 countries worldwide. It was first detected in 1980 at Tirat-Zvi, in eastern-northern Israel. In recent years, there has been an increasing concern over the spread of this weed in agricultural and non-agricultural habitats across the country. However, very little is known about the biology of *P. hysterophorus* and its variation among populations.

**Methods:**

Seeds collected from five locations across Israel were germinated and plants were grown in pollen-proof cages under uniform conditions to produce the progeny populations. Spatial parameters, weight and germination under different environmental conditions were recorded for field and progeny populations.

**Results:**

Seeds originating from field populations were significantly smaller and lighter than seeds of the progeny populations. Germination occurred in the range of 10°C to 30°C ( $T_{o}$ ranges from 19°C to 22.3°C, $T_{b}$ ranged from 9°C to 15°C, $T_{c}$ ranged from 24 ℃ to 30.5°C), depending on generation and population. A water potential-based model was developed to estimate germination under different soil water content using specific parameters ( b - slope, d - upper limit, e - infliction point). The model suggests a correlation between germination and water potential. Indeed, reduced germination was recorded for the lower water potentials especially for the progeny populations. Spatial parameters, weight and germination under different environmental conditions were recorded for field and progeny populations.

**Discussion:**

We identified differences in seed size and weight, germination under different temperatures, and osmotic potential among *P. hysterophorus* Israeli populations. Differences across generations may arise due to the transgenerational effects. Our results, may shed light on the germination abilities of *P. hysterophorus* populations and provide vital insight into understanding the invasive capabilities of this highly noxious weed.

## 1 Introduction

Invasive species are transported mainly via import-export trade (Levine et al., 2003). Climate change has enhanced invasive species spread range and adaptive ability, whose impact contributes to biodiversity loss and the deterioration of ecosystem services (Pyšek and Richardson, 2010). Noxious weeds are undesirable plants within the agricultural ecosystem (Oerke, 2006). and invasive weed species cause damage to the local flora and fauna, leading to significant economic and agricultural losses of up to almost $120 billion a year (Owen, 1998; Pimentel et al., 2005).

*Parthenium hysterophorus*, a noxious invasive weed of the *Asteraceae* family, originates from the countries surrounding the Gulf of Mexico (Rollins, 1950). While in natural habitats, *P. hysterophorus* grows as an annual plant, in invaded areas, it may develop as a perennial plant (Mahmoud et al., 2015). This species is an aggressive invasive weed threatening ecological and agricultural ecosystems around the globe (Adkins and Shabbir, 2014). In natural ecosystems, it causes a reduction in species richness and biodiversity mainly due to the large number of seeds it produces, which changes the seed bank ratio (Cowie et al., 2022). For example, *P. hysterophorus* was found to decrease savanna plant diversity and richness in South Africa (Cowie et al., 2022) and grassland in Australia (Nguyen et al., 2017). In India, extensive natural land where *P. hysterophorus* has invaded can no longer be used for agricultural purposes (Sushilkumar and Ray, 2010). *P. hysterophorus* can develop in a wide range of climatic conditions, infesting a wide range of countries with various cropping systems (Navie et al., 1996; Tamado et al., 2002; Kohli et al., 2006; Mcconnachie et al., 2011). This ability might have assisted in its widespread invasion of over 50 countries worldwide (Adkins and Shabbir, 2014; Safdar et al., 2015).

*P. hysterophorus* reproduction occurs by seeds, and the flowers are hermaphrodites. Each flower can produce five seeds with up to 15-20 thousand seeds per plant (Masum et al., 2013). *P. hysterophorus* seeds germinate under various temperatures and their germination is limited mainly by the relative soil humidity (Adkins and Shabbir, 2014). The seeds are spread by wind, water, animals, agricultural tools, and vehicle wheels. A wider spread, local and global, is through contaminated livestock and animal feed (Adkins et al., 2019).

*P. hysterophorus* is classified as a quarantine plant species, thus, any infested produce arriving in Europe should be eradicated (List, 2022). It is also known as a secondary host for pests and diseases circuitously harming the crop. For example, *P. hysterophorus* was documented as a host for *Phenacoccus solenopsis* (Arif et al., 2002), the bacteria *Pseudomonas solanacearum* (Kishun and Chand, 1987), and the Tomato yellow leaf curl virus (Govindappa et al., 2005). Furthermore, *P. hysterophorus* exerts an allergenic effect on humans and animals and may cause various inflammatory diseases. A high percent of the human population exposed to *P. hysterophorus* may develop an allergenic response (Towers and Rao, 1992; Kohli et al., 2006), including 11 identified variants of skin dermatitis (Lakshmi and Srinivas, 2012). Additionally, allergies, including asthma, bronchitis, and hay fever, may be caused by contact or the inhalation of airborne plant particles (Kohli et al., 2006).

*Parthenium hysterophorus* is a highly adaptive weed species that grows and develops under a wide range of environmental conditions (Adkins and Shabbir, 2014). The large number of seeds produced by *P. hysterophorus* and its phenotypic plasticity are most likely among the main reasons for its high invasion capabilities, as these characteristics may hasten its establishment in the invaded area (Rathee et al., 2021). Accordingly, the vast spread of invasive species is attributed to the production of a high number of small seeds (Daehler, 2003). Phenotypic flexibility is also a common trait for invasive species, which allows the plant to present multiple phenotypes for the same trait, depending on the environmental conditions in the invasion region (Rathee et al., 2021).

Differences in seed characteristics exist not only among species but also within each species. Both environmental factors and genetic traits may influence this variation (Baskin and Baskin, 1973; Benowicz et al., 2000). For instance, a sunflower (*Helianthus annuus*) crop grown in multiple locations across Pakistan showed differences in seed weight, which was attributed to the dissimilar environmental conditions at the different sites (Ahmad, 2001). Another study focusing on switchgrass (*Panicum virgatum*) looked at two biotypes and found that genetic factors and precipitation at each location had a larger influence than competition on the final seed weight (Boe, 2007).

Studying the germination and emergence abilities of invasive species is crucial to understanding their invasiveness potential and adaptation to new habitats. These biological traits are vital in this global climate change, with rising temperatures and CO_2_ levels predicted to increase the range and effects of invasive plant species (Clements and DiTommaso, 2011). Modeling germination under different environmental conditions can assist in predicting plant growth ranges and, therefore, may aid in containing and preventing their spread. In previous studies modeling invasive plant germination, one study focusing on *Solanum elaeagnifolium* temperature-based model presented similar germination patterns across populations while also presenting a wide range for germination (Kapiluto et al., 2022), while another study on *Amaranthus palmeri* showed different germination capabilities between populations assessed using a hydrothermal model (Matzrafi et al., 2021a).

Predicting plant development is done using models relating plant development and environmental factors, such as temperature, water potential, CO_2_ levels, and salinity (Ritz et al., 2013; Mesgaran et al., 2017). It is common to describe seed germination using non-linear regression, for example, using Log-logistic or Weibull distribution. Using these statistical distributions, along with a Time-to-event analysis, and taking into consideration the uncertainty of the documented event helps predict germination rates at different percentiles along with the maximal germination rate with greater accuracy (Onofri et al., 2022).

**Figure 1:**
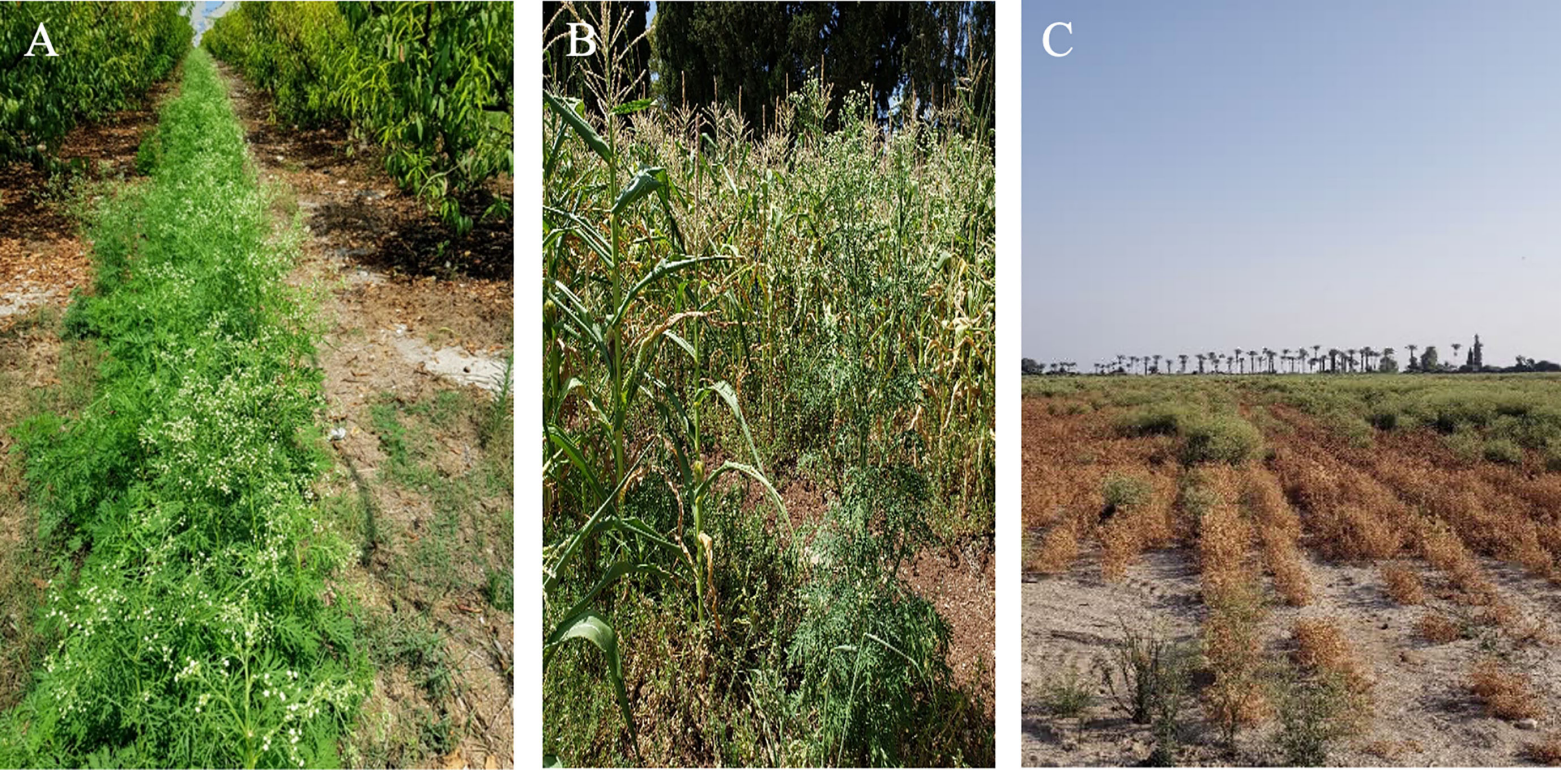
P*. hysterophorus* infestation in agricultural systems in Israel: orchard **(A)**, corn field **(B)** & chickpea field **(C)**.

In Israel, *P. hysterophorus* was first detected in 1980 at Tirat-Zvi (Dafni and Heller, 1982; Yaacoby, 2013) located in the eastern-northern area of the country. In recent years, there has been an increasing concern about the spread of this weed in agricultural and non-agricultural habitats across Israel. *P. hysterophorus* disrupts crops, such as corn, sorghum, chickpea, and watermelon, along with various orchards growing mainly between the crop and tree lines (**Figure 1**) (Matzrafi et al., 2021b).

This study aimed to describe seed-specific phenotypic variation among *P. hysterophorus* populations collected across Israel. Here, we studied the spatial parameters (area and perimeter) of the seeds, seed weight, and cardinal parameters for seed germination under various environmental conditions (temperature and water potential). These parameters were studied among populations and between generations (field and progeny populations).

## 2 Materials and methods

### 2.1 The seed collection

The seed collection of five *P. hysterophorus* populations was based on previous mapping conducted by Matzrafi et al. (2021b). Populations were chosen to represent a wide range of climatic and geographical zones in Israel. During the summer of 2019 (July-September), ripe inflorescences were collected from five locations in northern Israel: Amikam, Dgania Beit, Ha’hotrim, Newe Ya’ar, and Tirat Zvi. Habitats are differentiated by their land use (field crops, orchards, and roadsides) and environmental conditions, as specified in **Table S1**. At each location, inflorescence was collected haphazardly from 40 mature plants. At each site, all the collected inflorescences were hand-thrashed, seeds were bulked, and defined as populations, according to Silvertown and Charlesworth (2009).

### 2.2 Progeny populations reproduction

We have created the progeny populations using seeds collected in the field. Five-ten seeds were sown in 2L pots filled with planting soil (Ram 6, Tof Maron Golan) and placed in a climate-controlled greenhouse (20/25 C, day/night) at Newe Ya’ar Research Center. After germination, seedlings were diluted to one plant per pot. Once the bolting stage started (i.e., *P. hysterophorus* phenological growth stage), 30 plants of each population were placed in pollen-proof cages to prevent cross-pollination between populations. At the end of the season, ripe inflorescences were collected and hand-thrashed to create a seed bulk defined as the progeny population for each location. Seeds of these populations will be used for further germination experiments.

### 2.3 Spatial parameters and seed weight

To evaluate the spatial parameters, 100 seeds from each population were photographed using an electronic microscope (Hirox Europe, RH 2000) equipped with a digital camera (CMOS high-definition sensor) and a zoom lens (MXB-050Z 50-400x {20-800x}). A built-in image analysis software (RH 2000) was used to record the spatial parameters based on surface area (radius, area, diameter, perimeter) for each seed.

For seed weight, 1000 seeds were counted in 10 replicates of 100 seeds and were weighted on a micro-scale (Mettler-Toledo GmbH, WXTE).

### 2.4 Seed germination tests

Seeds were disinfected according to the protocol used for Broomrape (*Orobanche* and *Phelipanche* spp.) seed disinfection (Eizenberg et al., 2013). All trials were conducted in growth chambers (Pol-Eko-Aparatura incubator ISO:9001, 2008), and germination was tested under dark conditions as previously described by Matzrafi et al. (2021b). Each chamber was equipped with a temperature data logger (HOBO®, data logger; Onset Computer Corporation, Bourne, MA, USA). Germination was recorded once a day, at a set time, for 14 days.

### 2.5 Development of a temperature-based germination model

For each treatment (temperature), seeds were placed in a 9-cm petri dish lined with filter paper (Whatman® Grade 2; Sigma-Aldrich) and sealed with Parafilm® to reduce water loss from the dishes. Germination was assessed for six constant temperatures: 10, 15, 20, 25, 30, and 35 °C. Each population had four replicates (petri dishes) with 20 seeds at each temperature. To develop the temperature-based model, several equations were used:

The first was a log-logistic sigmoidal equation with five parameters –

(A)


$$f\left(x\right)=c+\frac{d-c}{{\left(1+exp\left(b\left(log\left(x\right)-\log\left(e\right)\right)\right)\right)}^{f}}$$

Parameters shown in this equation are the slope in the inflection point ( b ), upper limit ( d ), lower limit ( c ), and infliction point ( e ). The infliction point describes where 50% of maximal germination has accrued. When the value of f equals 1 the equation is symmetrical, when different, it is non-symmetrical. Using this equation, the value of $t_{50}$ was extracted, the time for 50% of maximal germination or the time for the e parameter value for each trial repetition. $t_{50}$ was converted into germination rate ( $GR_{50}$ ) for that percentile using the following equation –

(B)


$$GR_{g}=\frac{1}{t_{g}}$$

When g refers to the percentile used.

We also used the equation for the polynomial temperature effect model (Mesgaran et al., 2017) with three parameters-

(C)


$$GR=\frac{max\left(T,T_{b}\right)-T_{b}}{\theta_{T}}\left[1-\frac{\min\left(T,T_{c}\right)-T_{b}}{T_{c}-T_{b}}\right]$$

Parameters in this equation express the basic temperature ( $T_{b}$ ), maximum temperature ( $T_{c}$ ), and the thermal time for germination ( $\theta_{T}$ ). Using the values of T and $GR_{50}$ of each repetition in equation C, all the above parameters are given.

Since this curve is symmetrical, calculating the optimal temperature values was derived from the following equation–

(D)


$$T_{o}=\frac{T_{c}-T_{b}}{2}+T_{b}$$

Data analysis was performed using R (version 3.3.2, i386) in the RStudio (version 1.3.1056) integrated environment using the *drm*() function (Onofri et al., 2018) and *GRT.M*() function for the polynomial temperature effect for a cardinal model (Team, R. D. C, 2020).

### 2.6 Water potential-dependent germination model

Experiments were conducted using the protocol described in Matzrafi et al. (2021a). Solutions were prepared by dissolving the appropriate amounts of polyethylene glycol (PEG) 8000 (Sigma-Aldrich, St. Louis, MO) in deionized water. Filter papers (Whatman® Grade 2; Sigma-Aldrich) were soaked in each solution containing the desired water potential for 12 h prior to the experiment. The water potential of each solution was tested using a Wescor Vapro osmometer (Wescor, Logan, UT, USA). This experiment used five concentrations of PEG solution to create water potentials of -0.2, -0.4, -0.6, -0.8, and -1 Mega pascal (MPa) at a temperature of 20°C. Each population had four replicates with 20 seeds for each water potential.

### 2.7 Statistical analysis

JMP Pro (Version 16, SAS institute Inc) statistical software was used for the analysis of variance (ANOVA) followed by comparing means by t-test for each pair or Tukey-HSD (Honestly significant difference) for multiple pairs.

Model selection was made using the Akaike information criterion (AIC) value (Akaike, 1974), considering the complexity and accuracy of each model according to the number of parameters. The equation to calculate the value of AIC is-

(E)


$$AIC=2m+n\ln\left(\frac{RSS}{n}\right)+\frac{2*m\left(m+1\right)}{n-m-1}$$

Where m is the number of parameters, n is the number of replicates, and RSS is the residual sum of squares.

A sigmoidal Log-logistic three parameter equation was used to analyze the water potential-dependent germination model.

(F)


$$f\left(x\right)=\frac{d}{1+\exp\left(b\left(\log\left(x\right)-\log\left(e\right)\right)\right)}$$

The lower limit is set at 0, parameters are the slope ( b ), upper limit ( d ), and infliction point or where 50% of maximal germination has accrued ( e ).

## 3 Results and discussion

**Figure 2:**
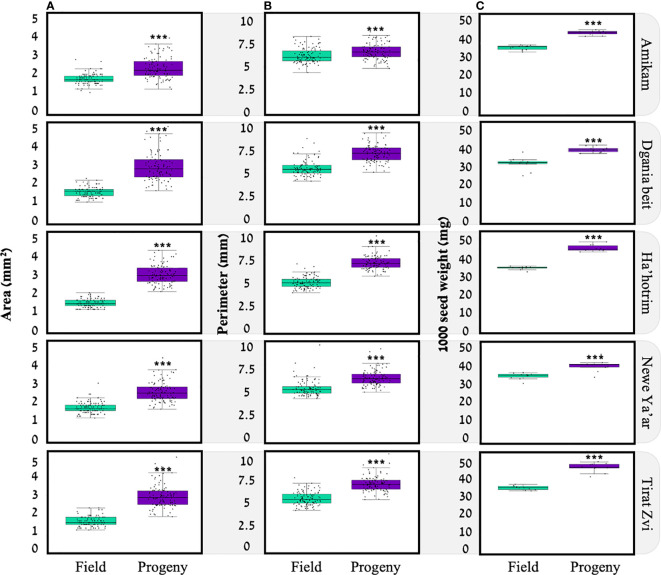
Spatial parameters for seeds of five *P*. *hysterophorus* field and progeny populations; **(A)** surface area (n=100), **(B)** seed perimeter (n=100), **(C)** 1000 seed weight (n=10). *** statistically significant by *t-test p<0.05*.

In this study, we examined seed area, perimeter, and weight (**Figure 2**). We found that, for each location, seeds from the progeny populations were larger compared to seeds of the field populations. Indeed, several studies showed a link between seed parameters and progeny plant development to growth conditions under which the mother plant was grown (Matzrafi et al., 2021a). In our experiment, seeds of the progeny populations were produced under optimal conditions so that we can assume maximum resource allocation towards seed production. However, we can also assume this was not always the case for field population seeds, as the environmental conditions were quite different among locations where seeds were collected, e.g., water availability and temperature (**Table S1**). For *Amaranthus retroflexus*, plants grown under water deficit produce heavier seeds than those grown under optimal conditions,(Chadoeuf-hannel and Barralis, 1982) as is the case for *A. palmeri* (Matzrafi et al., 2021a). A study examining four members of *Polygonum* spp. showed that three (*P. persicaria*, *P. cespitosum*, *P. lapathifolium*) had larger achenes when plants were grown under deficit irrigation, while plants grown under well-watered conditions had smaller achenes. However, one *Polygonum* species, *P. hydropiper*, showed a similar trend to our study of larger seeds produced under ample water (Sultan, 2001). These results suggest that the effect of water availability on seed traits is species-specific.

**Figure 3:**
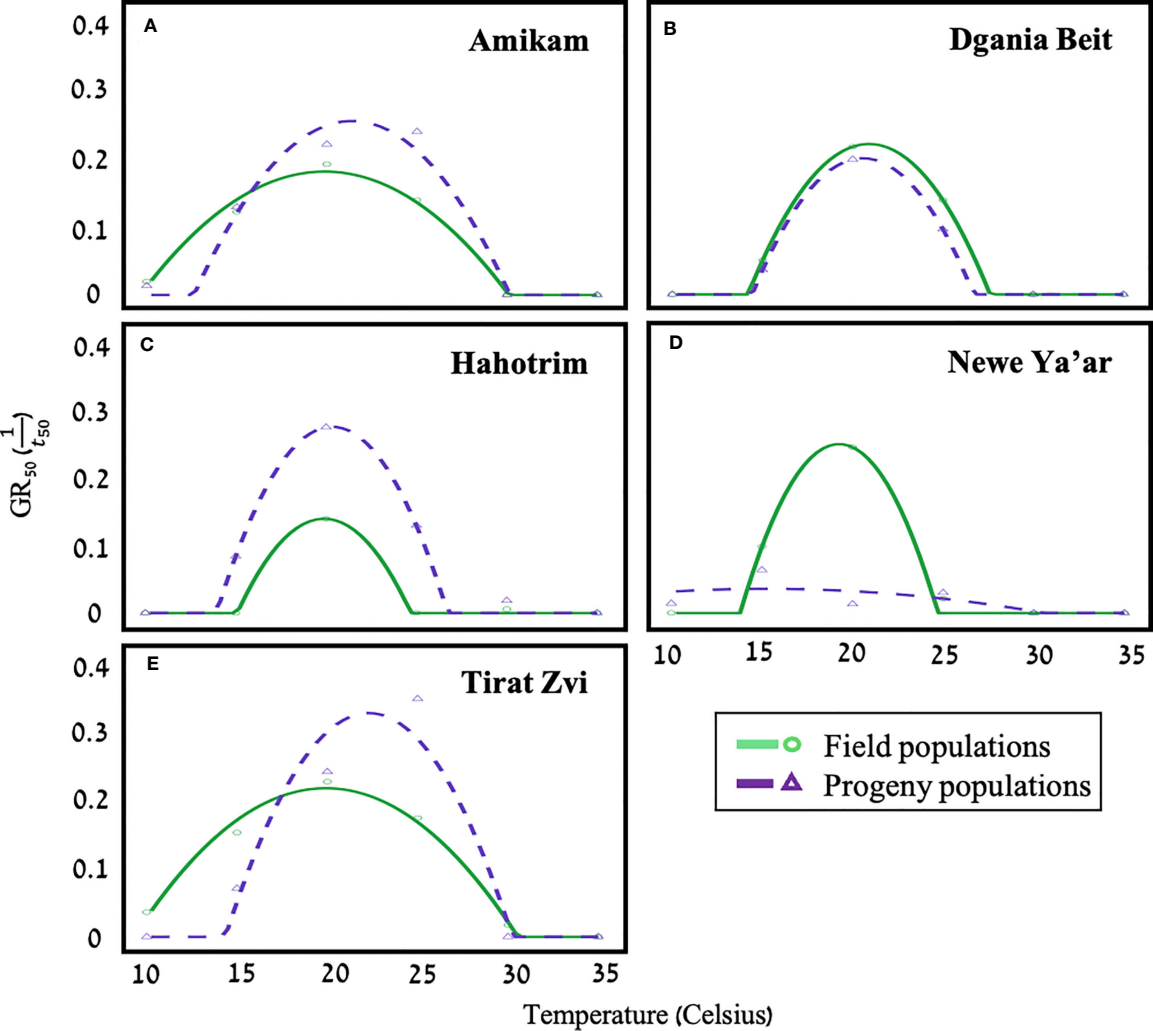
A polynomial temperature effect model estimating the germination rate (GR_50_) at different temperatures for five field (solid line; dots) and progeny (dashed line; triangles) populations of *P. hysterophorus* [**(A)** Amikam, **(B)** Dgania Biet, **(C)** Hahotrim, **(D)**- Newe Ya’ar, **(E)** Tirat Zvi]. Model fit using polynomial temperature effect equation (equation B, materials, and methods), *n*=20.

For seed weight, among field populations, Dgania Beit seeds show the lowest average weight (30.95 ± 1.13; **Figure 3**). In previous studies, a connection was found between seed weight and plant growth temperature during seed development, when high temperatures occur at the seed production stage, smaller seed weight was recorded and vice versa (Fenner, 1991; Nasehzadeh and Ellis, 2017). Indeed, when examining the average temperature at the different locations (**Table S1**), Dgania Beit and Tirat Zvi had higher average temperatures than other sites during the seed collection year. However, although high temperatures are typical in Beit She’an Valley, where Tirat Zvi population seeds were collected (Cabra-Leykin et al., 2020), Tirat Zvi did not have seeds with a lower weight. Tirat Zvi seeds were collected from a date orchard, a main crop in the Beit She’an Valley, where high tree canopy may reduce the temperature by 1-2°C (Bowler et al., 2010; Coutts et al., 2016), and thus, the temperature may exert a lower effect on seed weight. Differences in seed weight may be attributed to other environmental conditions; however, further research is needed to understand these effects. Alternatively, the differences in seed size within generations and among locations could be related to the ratio between seed size and number. Since a given amount of resources are available for seed production, if a plant produces heavier seeds, the number of seeds may decrease and vice versa (Leishman, 2001). Further studies in our lab are focused on understanding the seed amount/size correlation in *P. hysterophorus*.

To examine the G x E (e.g., genetic, environment) related influences, progeny seeds were reproduced under uniform conditions for each population. By comparing the seeds of the progeny populations, we found that Amikam, Ha’hotrim, and Tirat Zvi populations had a higher average seed weight compared to Dgania Beit and Newe Ya’ar (**Table 1**). On the other hand, the field and progeny population from Dgania Beit had a lower average seed weight compared to all other populations (**Table 1**). Since seeds of Dgania Beit were produced under the same conditions as all other progeny populations, a genetic factor may be determining seed size in this population.

**Table 1:** Comparing 1000 seed weight (mg ± standard error) among populations of *P*. *hysterophorus* for field and progeny (for each generation separately).

Population	1000 Seed weight (mg)	
	Field	Progeny
Amikam	35.54 ± 0.4 A	43.58 ± 0.39 A
Dgania Beit	30.95 ± 1.13 B	38.48 ± 0.48 B
Ha’hotrim	34.17 ± 0.29 A	45.05 ± 0.56 A
Newe Ya’ar	35.08 ± 0.56 A	40.73 ± 1.02 B
Tirat Zvi	34.01 ± 0.38 A	45.11 ± 0.8 A

Different letters show statistically significant differences among populations of each generation according to Tukey HSD test *p>0.05.*

Models estimating germination serve as a critical tool in designing support systems to maximize the efficacy of herbicide application time and amount (Lati et al., 2011; Eizenberg et al., 2012; Cochavi et al., 2016; Reinhardt Piskackova et al., 2020). Temperature, water potential, light, and soil pH all impact the rate and speed of seed germination (Ritz et al., 2013; Mesgaran et al., 2017). We studied the effect of temperature and water availability on the germination capabilities of seeds from five different field and progeny *P. hysterophorus* populations (**Figure 3**). The model developed in this study shows that the optimal temperature for seed germination for the field population is 19.5°C - 21°C and for progeny populations 20°C - 22.5°C. However, for the Newe Ya’ar progeny seeds, the optimal temperature for germination was 17.5°C (**Table 2**). Seeds of Dgania Beit field and progeny populations presented the same optimal temperature for germination. Compared to other field populations, Dgania Beit seeds required the lowest temperature for seed germination, while their progeny populations required the highest (**Table 2**). Therefore, we can assume that a genetic factor has a substantial impact on the germination of Dgania Beit field and progeny seeds. Even when grown under two different growth conditions, the optimal temperature for germination did not change, while other populations examined in this study did show differences between generations.

**Table 2:** Parameters of the polynomial temperature effect for germination of *P. hysterophorus* field and progeny populations under different temperatures.

Population/Generation	Parameter	T c		T b		$\theta_{T}$		$T_{o}$	
		Field	Progeny	Field	Progeny	Field	Progeny	Field	Progeny
Amikam	Estimate	30.101	30.160	9.644	12.619	28.758	17.485	19.873	21.39
	Std. Error	0.945	0.606	0.989	1.003	4.752	2.630		
	p-value	0.0001	0.0001	0.0001	0.0001	0.0001	0.0001		
Dgania Beit	Estimate	27.611	26.702	14.190	14.240	15.379	15.554	20.901	20.561
	Std. Error	1.053	0.800	0.583	0.545	3.108	3.099		
	p-value	0.001	0.0001	0.0001	0.0001	0.0001	0.0001		
Ha’hotrim	Estimate	24.364	26.735	15.032	13.939	17.083	11.904	19.698	20.337
	Std. Error	109.488	0.484	197.769	0.385	397.039	1.445		
	p-value	0.825	0.0001	0.940	0.001	0.966	0.0001		
Newe Ya’ar	Estimate	25.254	28.486	13.707	6.583	11.968	128.474	19.48	17.535
	Std. Error	0.194	4.262	0.277	4.349	0.931	75.093		
	p-value	0.0001	0.0001	0.0001	0.138	0.0001	0.094		
Tirat Zvi	Estimate	30.546	30.242	9.285	14.351	24.757	12.292	19.916	22.296
	Std. Error	0.932	0.460	0.961	0.505	3.770	1.349		
	p-value	0.0001	0.0001	0.0001	0.0001	0.0001	0.0001		

$T_{c}$ - Maximum temperature for seed germination, $T_{b}$ - base temperature for seed germination, $\theta_{T}$ - thermal time for seed germination, $T_{o}$ - optimal temperature for germination.

Compared to their field population and all other populations, the Newe Ya’ar progeny population exhibited interesting results, hardly showing any germination (**Figure 3**). Yet, when seed viability was examined using triphenyl tetrazolium chloride (TTC), a high vitality rate was found (**Table S2**; 86.67%). A study conducted in India found that different environmental conditions at the time of seed development can lead to seed dormancy for *P. hysterophorus* seeds of the same population (Javaid et al., 2010). Therefore, the Newe Yaar population may be associated with a second biotype. Additionally, a study from Australia found two *P. hysterophorus* biotypes differentiated by their germination capabilities relating to their invasiveness. Researchers in this study examined germination under different environmental conditions and found that the Clermont biotype germinated better across all treatments compared to the Toogoolawah biotype (Navie et al., 1996). Further experiments should be aimed at elucidating the environmental conditions that reduce seed dormancy for seeds of the Newe Ya’ar progeny population and further examine the assumption of a second biotype.

**Figure 4:**
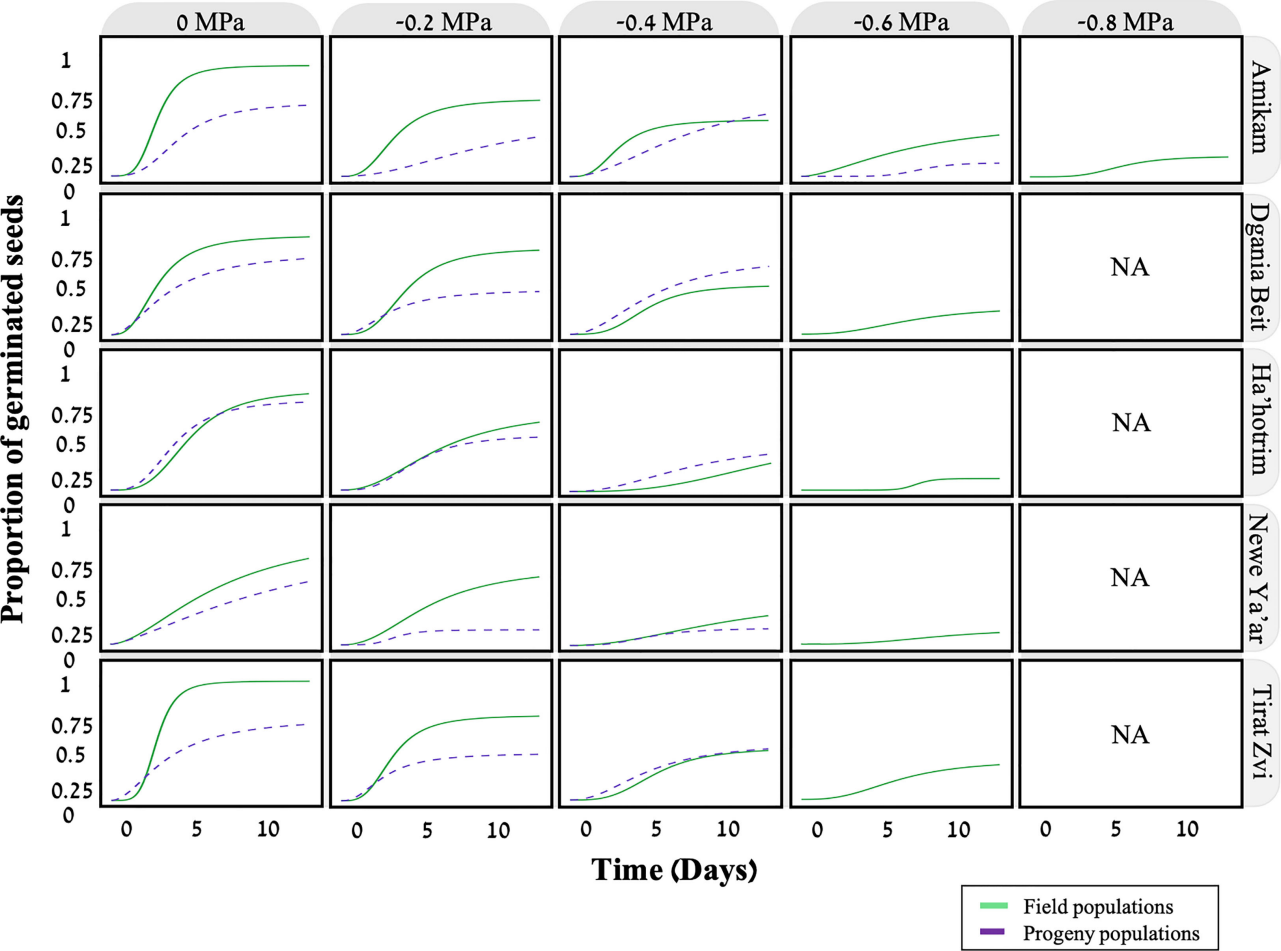
The effect of different water potentials (0, -0.2, -0.4, -0.6, -0.8, -1 MPa) on the germination of five field (solid line) and progeny (dashed line) populations of *P. hysterophorus*. Model fit using time-to-event nonlinear regression with Log logistic three-parameter curve (equation C, materials, and methods), *n*=20.

Aside from temperature, the soil water potential may also affect seed germination (Bradford, 2002). To this end, we examined the effect of five water potentials (-0.2, -0.4, -0.6, -0.8, -1 MPa) on seed germination from five field and progeny populations. According to the fitted model, there is a clear correlation between seed germination and water potential, with the germination rate decreasing in lower water potentials and none of the seeds germinating at -1MPa (**Figure 4**; **Table 3**). A previous study found a similar trend in the seed germination of two *P. hysterophorus* biotypes under various water potentials (Bajwa et al., 2018). For both field and progeny populations, seeds from the Amikam population germinated under lower water potentials compared to the other populations (-0.8 and -0.6 MPa, respectively). According to the Israeli soil survey (**Table S1**), the Amikam area is characterized by light brown rendzina soil with low water holding capacity (Dan and Koyumdjisky, 1963). This difference in water potential tolerance can result from a specific *P. hysterophorus* adaptation to the environmental conditions in the Amikam area. Since both field and progeny populations demonstrated this characteristic, we assume this is a genetic factor and not only environmental.

**Table 3:** Estimate of e parameter of the log logistic three-parameter equation for germination of *P. hysterophorus* field and progeny populations under different water potentials.

Population	Water potential (MPa)	0		-0.2		-0.4		-0.6		-0.8		-1	
		Field	Progeny	Field	Progeny	Field	Progeny	Field	Progeny	Field	Progeny	Field	Progeny
Amikam	Estimate	3.2091	4.7861	3.7437	10.1093	3.4324	8.4521	7.8968	8.4345	6.4035	NA	NA	NA
	Std. Error	0.1745	0.4462	0.3626	3.9087	0.3485	2.3645	3.5324	0.7336	0.8343	NA	NA	NA
	p-value	0.0001	0.0001	0.0001	0.0097	0.0001	0.0004	0.0254	0.0001	0.0001	NA	NA	NA
Dgania Beit	Estimate	3.2567	3.9684	4.5330	3.4675	5.2881	5.8723	7.8156	NA	NA	NA	NA	NA
	Std. Error	0.2823	0.6909	0.3729	0.6294	0.5325	1.2129	2.1504	NA	NA	NA	NA	NA
	p-value	0.0001	0.0001	0.0001	0.0001	0.0001	0.0001	0.0003	NA	NA	NA	NA	NA
Ha’hotrim	Estimate	5.4908	4.4788	6.8858	5.2324	14.4688	5.3854	8.1079	NA	NA	NA	NA	NA
	Std. Error	0.4133	0.3708	1.2960	0.5763	8.0280	0.7409	0.4214	NA	NA	NA	NA	NA
	p-value	0.0001	0.0001	0.0001	0.0001	0.0715	0.0001	0.0001	NA	NA	NA	NA	NA
Newe Ya’ar	Estimate	7.8070	11.9804	6.2658	3.8171	11.4744	5.5858	9.8777	NA	NA	NA	NA	NA
	Std. Error	2.3926	7.3413	1.1667	0.4816	6.9486	1.0259	4.4533	NA	NA	NA	NA	NA
	p-value	0.0011	0.1027	0.0001	0.0001	0.0987	0.0001	0.0265	NA	NA	NA	NA	NA
Tirat Tzi	Estimate	3.2111	3.9685	3.5827	3.0341	6.0480	5.5948	6.8887	NA	NA	NA	NA	NA
	Std. Error	0.1402	0.6909	0.2802	0.4624	0.6557	1.0813	1.2548	NA	NA	NA	NA	NA
	p-value	0.0001	0.0001	0.0001	0.0001	0.0001	0.0001	0.0001	NA	NA	NA	NA	NA

e - infliction point or where 50% of maximal germination has accrued.

NA, not applicable.

In a previous study conducted in Italy, germination was tested for *Aegilops geniculate* from three locations under different water potentials. A negative correlation was found between the germination rate at -1.7MPa and rainfall at each site, with the location with the lowest rainfall having the highest germination percentage (Orsenigo et al., 2017).

The other four populations examined in this study exhibited seed germination at water potentials as low as -0.6 MPa in field populations and -0.4 for progeny populations MPa (**Figure 4**). Our data show that seeds from progeny populations are more sensitive to low water potential than seeds from field populations. As previously mentioned, the mother plants of the progeny populations were grown under optimum conditions compared to plants growing under field conditions. In their study, Matzrafi et al. (2021a) compared seeds from plants grown under well-watered and water-deficit conditions. Seeds produced by well-watered plants showed lower germination rates at more negative water potentials than seeds from plants grown under water-deficit conditions. Another study in Chile tested the germination of *Lycopersicon chilense* (wild tomato) seeds showed similar results, with well-watered plants producing seeds sensitive to low water potentials (Maldonado et al., 2002). This may be explained as adaptation to extreme conditions faced by the mother plants affects the response of their progeny.

To conclude, in this study, we identified differences among populations and across generations of *P. hysterophorus* concerning seed size and weight, germination rate, and the response to different temperatures and water potentials. We suggest that this may be because of maternal effect caused due to exposure to various environmental conditions at different locations. Further research is needed to combine our results describing phenological traits with future genomic data. This may shed light on the origin of *P. hysterophorus* populations and serve as a key for understanding the invasive abilities of this highly noxious species.

## Data availability statement

The original contributions presented in the study are included in the article/**Supplementary Material**. Further inquiries can be directed to the corresponding author.

## Author contributions

SM, HE and MM all contributed to the current study and to writing the paper. All conceived and designed the study. SM collected and analyzed the data. All authors contributed to the article and approved the submitted version.
