# Supplementary material for: Variation in seed properties and germination capabilities among populations of the invasive weed Parthenium hysterophorus L. (Asteraceae)
Source: Front Plant Sci. 2023 Jul 27;14:1222366. doi: 10.3389/fpls.2023.1222366 (PMC10421661; doi:10.3389/fpls.2023.1222366)
Supplement: Supplementary file 1 [file Table_1.docx]

**Appendix**

**Table 1S.** Environmental conditions data for all five *P. hysterophorus* seed collection sites, at the year of collection (2019). Soil types were gathered from a soil group map of the Israeli Agricultural ministry; Temperature, relative humidity and rainfall were collected form the Israeli metrological services archives and open data source. ± stands for the SE.

**Table 2S.** Percentage of *P. hysterophorus* seed viability in field and progeny populations, *n*=20.

*Viability was assess using triphenyl tetrazolium chloride (TTC) viability test. In summary, 20 seeds of each population were placed in a 50ml tube and submerged in a 1% TTC solution. Tubes were placed for 48 hours in a 60C incubator. after the set time seeds were cut and visually tested for red coloring of the seed embryo. Only bright red colored seeds were classified as viable and pink to white colored seeds as non-viable.
